# Neuropilin-1 is upregulated by Wnt/β-catenin signaling and is important for mammary stem cells

**DOI:** 10.1038/s41598-017-11287-w

**Published:** 2017-09-08

**Authors:** Wei Liu, Ting Wu, Xiaobing Dong, Yi Arial Zeng

**Affiliations:** State Key Laboratory of Cell Biology, CAS Center for Excellence in Molecular Cell Science, Shanghai Institute of Biochemistry and Cell Biology, Chinese Academy of Sciences, University of Chinese Academy of Sciences, Shanghai, 200031 China

## Abstract

Wnt/β-catenin signaling is instrumental for the development of mammary gland and the properties of mammary stem cells (MaSCs). The Wnt signaling downstream effectors that engage in regulating MaSCs have not been extensively studied. Here, we report that Neuropilin-1 (Nrp1) expression is induced by Wnt/β-catenin signaling in MaSCs, and its function is critical for the activity of MaSCs. Nrp1 is particularly expressed in MaSCs that are marked by the expression of Protein C Receptor (Procr). Knockdown of Nrp1 by shRNA diminishes MaSCs’ *in vitro* colony formation and *in vivo* mammary gland reconstitution ability. Similar results are seen when antagonizing Nrp1 using a dominant negative peptide. In genetic experiments, deletion of Nrp1 results in delay of mammary development. In addition, knockdown of Nrp1 inhibits *MMTV-Wnt1* tumor growth in xenograft. Our data demonstrate that Nrp1 is critical for mammary development and tumorigenesis, revealing new insights into MaSC regulation and targeting stem cells in treatment of breast cancer.

## Introduction

The mammary gland is an epithelial organ, with a tree-like pattern of ductal networks. The majority of mammary development occurs postnatally. At the onset of puberty at around 3 weeks of age in mice, in response to ovarian hormones, the preexisting rudimentary ductal tree rapidly expands and extends across the fat pad, occupying the whole mammary fat pad by approximately 7 weeks of age^1^. Highly elongated basal cells and cuboidal luminal cells compose the two main cellular lineages of the nulliparous and non-pregnant mammary gland. The basal cell population (Lin^−^, CD24^+^, CD29^hi^/CD49f^hi^) is able to generate new mammary glands in transplantation assays, thus representing a mammary stem cells (MaSCs)-enriched population^2, 3^. More recently, study from our lab reveals a more refined MaSC population that is marked by the expression of Protein C Receptor (Procr). Procr^+^ MaSCs are composed of about 3–8% of total basal cells depending on the genetic background. Procr^+^ MaSCs have the highest reconstitution efficiency in transplantation assays compared to total basal cells and other known basal subpopulation^4^.

Wnt/β-catenin signaling has been implicated in almost all stages of mammary development and is instrumental for MaSC self-renewal and expansion activities (reviewed in refs 5–7). Studies have directly addressed Wnts as niche factors for MaSCs^8, 9^. In 3D Matrigel cultures, addition of Wnt3A or Wnt4 proteins to MaSC-enriched basal cell culture can maintain stem cell properties and promote MaSC expansion. The retention of stem cell properties is demonstrated by the ability of the cultured cells to efficiently reconstitute mammary glands in transplantation^8, 9^. In an attempt to identify Wnt targets specifically expressed in MaSCs, microarray analysis of cultured MaSC-enriched basal cells was performed, leading to the discovery of the MaSC specific surface marker Procr^4^.The microarray analysis also suggests other new Wnt downstream target genes in mammary epithelial cells, which are potentially critical for the activities of MaSCs.

Neuropilin-1 (Nrp1) is a single-pass transmembrane glycoproteins, with a small cytoplasmic domain and multiple extracellular domains^10^. Nrp1 binds to a variety of ligand families, functioning as co-receptors in a complex with other transmembrane receptors^11^. The class 3 semaphorins (SEMA3) and vascular endothelial growth factor (VEGF) family are well established ligands for Nrp1^12, 13^. Evidence has revealed that the Nrp1 also interacts with other growth factors^11^. Nrp1 and it close family member Nrp2 are mostly known for the regulation of cell motility, particularly with respect to neural and vascular development^12–17^. Nrp1 may play a role in epithelial cells as well. Robust Nrp1 expression has been found in human epithelial tumor cells derived from lung, breast, prostate, pancreatic, and colon carcinomas^11^. Nrp1 has also been implicated in the migration and survival of breast cancer cells^18–20^, however its potential role in MaSCs and in normal mammary development remains elusive. In this study, we identified Nrp1 as a novel target of Wnt/β-catenin signaling. We showed that the expression of Nrp1 is enriched in Procr^+^ MaSCs, and that Nrp1 plays an essential role in MaSC property maintenance and *MMTV-Wnt1* mammary tumor growth.

## Results

### Nrp1 is upregulated by Wnt signaling in Procr^+^ MaSCs

**Figure 1:**
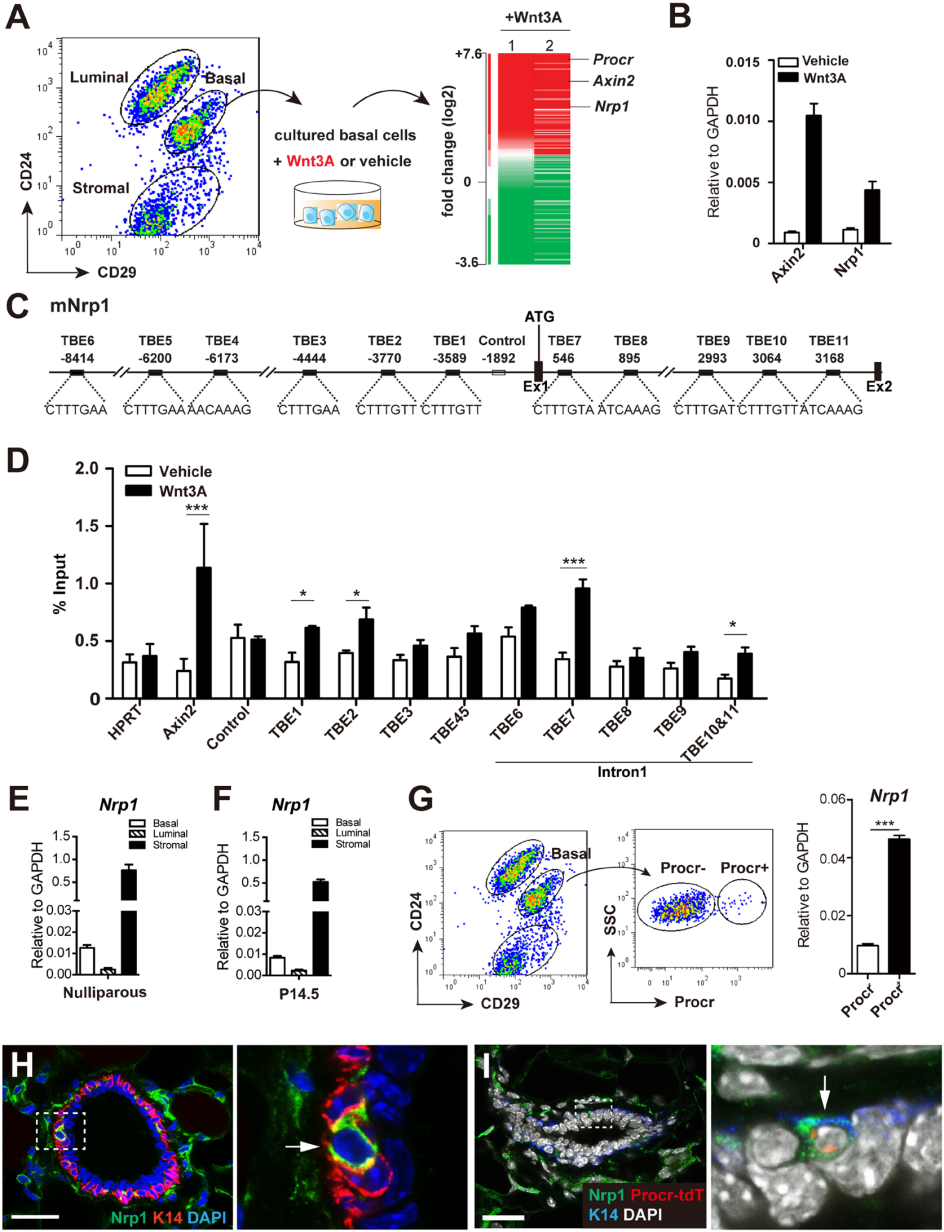
Nrp1 is upregulated by Wnt signaling in MaSCs. (**A**) Mammary basal cells were FACS-sorted from 8-week-old nulliparous mammary gland and cultured in 3D Matrigel in the presence of Wnt3A protein or vehicle. Microarray analysis of the cultured cells indicated that Nrp1 was upregulated with Wnt3A treatment. 1 and 2 represented two independent experiments. (**B**) qPCR analysis validating the increased expression of *Nrp1* in Wnt3A treated cells. *Axin2* serves as a positive control. (**C**) Schematic illustration of the promoter and first intron of mouse *Nrp1*. TCF-binding elements (TBEs) and negative control region are indicated. Ex, Exon; Int, Intron. (**D**) ChIP-qPCR analysis with anti-β-catenin antibody. qPCR results were normalized to HPRT. Axin2 promoter region with known effective TEB serves as positive control. (**E**,**F**) qPCR analysis of nulliparous mammary gland (**E**) and pregnant day 14.5 (P14.5) (**F**) indicating that *Nrp1* expression is higher in basal cells compared to luminal cells, and it has the highest expression in stromal cells. (**G**) qPCR analysis of FACS-isolated Procr^+^ and Procr^−^ basal cells from nulliparous mammary gland indicating that Nrp1 is mainly expressed in Procr^+^ basal cells. Data are pooled from three independent experiments, are presented as mean ± SEM. ***P < 0.001. (**H**) Immunohistochemistry indicating that Nrp1 is expressed in a subpopulation of basal cells (arrow). Basal cells are marked by the expression of Keratin 14 (K14) in red. (**I**) Immunohistochemistry indicating Nrp1 expression (green) in a tdTomato^+^ Procr-expressing cell (Procr-tdT). Scale bar, 20 μm.

Previous studies established *in vitro* culture system in which MaSC properties can be maintained using purified Wnt proteins^8^. In this culture system, mammary basal cells (Lin^−^, CD24^+^, CD29^hi^) were isolated using fluorescence-activated cell sorting (FACS) and cultured in 3D Matrigel in the presence or absence of Wnt3A proteins^4^. Microarray was performed using the cultured cells to identify downstream effectors of Wnt signaling in regulating MaSCs (Fig. 1A). Among the candidates whose expressions were increased in the presence of Wnt3A, which included *Axin2* and *Procr*, we identified *Nrp1* (Fig. 1A). Quantitative PCR (qPCR) confirmed that *Nrp1* expression is upregulated by Wnt3A treatment (Fig. 1B). Upregulation of *Axin2* in this condition served as a positive control (Fig. 1B).

Sequence analysis revealed six putative TCF-binding elements (TBEs) at the promoter and distal region and five putative TBEs at the first intron of *Nrp1* (Fig. 1C). To test whether the β-catenin can directly associated with*Nrp1*, we performed chromatin immunoprecipitation (ChIP) assays. We used β-catenin antibody to immunoprecipitate chromatin DNA in mouse mammary cells and examined specific binding of the following: 9 separate fragments containing the TBE of *Nrp1*; a fragment without TBE at *Nrp1* promoter region which acted as a negative control; and a fragment with one TBE at the *Axin2* promoter as a positive control. As shown in Fig. 1D, the β-catenin antibody immunoprecipitated TBE1 and TBE2 at the promoter, and TEB7, TBE 10/11 at the intron, whereas no precipitation was observed in the negative control. These data suggest that Nrp1 is a target of the Wnt/β-catenin pathway in mammary cells.

To investigate the expression pattern of Nrp1 in the mammary gland, we isolated basal (Lin^−^, CD24^+^, CD29^hi^), luminal (Lin^−^, CD24^+^, CD29^lo^) and stromal cells (Lin^−^, CD24^−^) from 8-week old nulliparous mice, and analyzed *Nrp1* levels by qPCR. We found that in the epithelial compartment, *Nrp1* is markedly higher in basal cells than that in luminal cells, while it displays the highest level in stromal cells (Fig. 1E). Similar expression patterns were observed in mammary gland at pregnant day 14.5 (Fig. 1F). The expression of *Nrp1* was further examined in basal cells in higher resolution. MaSCs (Lin^−^, CD24^+^, CD29^hi^, Procr^+^) and the other basal cells (Lin^−^, CD24^+^, CD29^hi^, Procr^−^) were isolated from mature nulliparous female mice. We found that *Nrp1* is expressed at a higher level (4.5 fold increase) in Procr^+^ MaSCs by qPCR analysis (Fig. 1G). Immunofluorescent staining in mammary gland section confirmed the Nrp1 expression pattern. We observed that a small population of basal cells is Nrp1^+^ (5.48 ± 1.93%), whereas no Nrp1^+^ cells are found in luminal cells (n = 1,000 basal or luminal cells) (Fig. 1H). Of note, a large number of Nrp1^+^ cells were seen in the stromal compartment (Fig. 1H), which is consistent with the qPCR results. To investigate whether Nrp1^+^ basal cells coincide with Procr^+^ MaSCs, we utilized Procr-tdTomato knock-in (Procr^CreERT2-IRES-tdTomato^) mouse model, in which tdTomato serves as a reporter for Procr expression^4^. Immunostaining indicated that there is a substantial overlap of Nrp1 and tdTomato expression in basal compartment, in which about 34% of Procr^+^ basal cells were Nrp1^+^ (116 out of 348 Procr^+^ basal cells) (Fig. 1I). This can be explained by the potential heterogeneity of the Procr^+^ basal cells. In contrast, very few Procr^−^ basal cells were Nrp1^+^ (11 out of 838 Procr^−^ basal cells, 1.3%). Together, these data suggest that in the epithelial compartment, Nrp1 is highly expressed in Procr^+^ MaSCs.

### Nrp1 is critical for MaSC activities *in vitro* and *in vivo*

**Figure 2:**
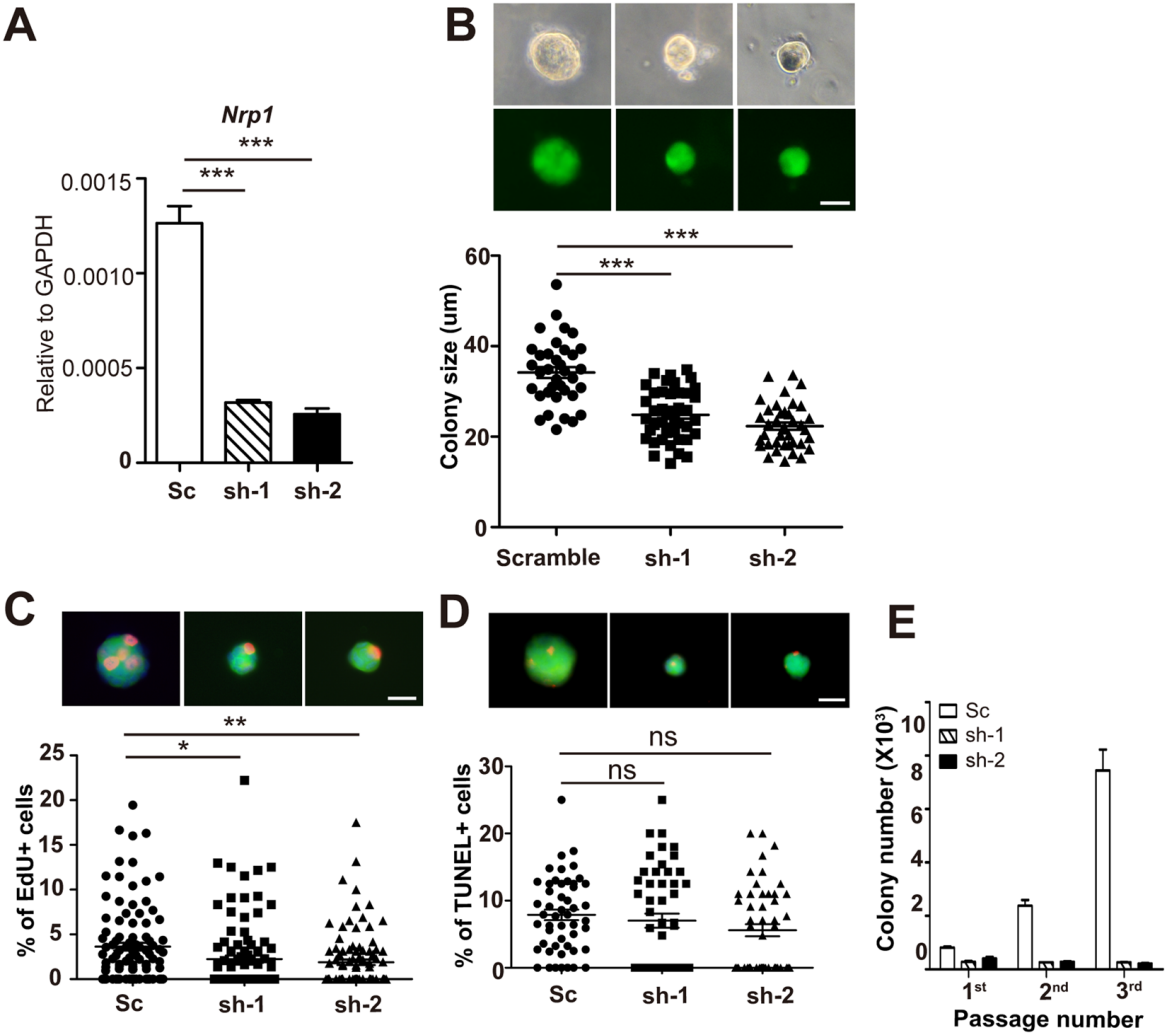
Nrp1 expression is important for MaSC colony formation *in vitro*. (**A**) qPCR analysis validating the *Nrp1* knockdown efficiency of two independent shRNAs. Data are pooled from three independent experiments, are presented as mean ± SEM. ***P < 0.001. (**B**) Representative images of colonies upon infection with scramble or *Nrp1* shRNAs (sh-1, sh-2) viruses (top panels), and quantification of colony sizes at cultured day 6 (bottom panels). Each dot represents one colony. Scale bar, 20 μm. Data are presented as mean ± SEM. ***P < 0.001. (**C**) Representative images of EdU labeled basal colonies (top panels). EdU^+^ cell percentages (EdU^+^ cells/DAPI^+^ cells in each colony) were quantified at day 6 (bottom panel). Each dot represents one colony. Scale bar, 20μm. Data are presented as mean ± SEM. *P < 0.05, **P < 0.01. (**D**) Representative images of TUNEL staining of basal colonies (top panels). TUNEL^+^ cell percentages (TUNEL^+^ cells/DAPI^+^ cells in each colony) were quantified at day 6 (bottom panels). Each dot represents one colony. Scale bar, 20μm. Data are presented as mean ± SEM. (**E**) Serial passage of basal colonies after lentiviral infection with scramble or Nrp1 shRNAs. The colony numbers of each passage were quantified.

Next, we investigated the role of Nrp1 in MaSCs *in vitro*. Isolated basal cells from nulliparous mice were infected with lentivirus containing GFP tagged Nrp1 shRNA. Infected cells were placed in 3D Matrigel culture and the formed colonies were visualized by GFP. To minimize potential off-target effect, two independent sh-RNAs were constructed for *Nrp1* knockdown. The knockdown efficiency of sh-Nrp1 was confirmed by qPCR analysis (Fig. 2A). We observed that knockdown of Nrp1 results in reduction of colony sizes (Fig. 2B), suggesting a decreased proliferation. Reduced EdU incorporation in Nrp1 knockdown cells corroborated with the idea (Fig. 2C). In addition, TUNEL staining suggested no discernable difference in cell death upon *Nrp1* knockdown (Fig. 2D). Furthermore, we found that colony numbers were decreased in serial passages when *Nrp1* expression is inhibited (Fig. 2E), suggesting that the numbers of stem cell-like cells were decreased in each passage. These data suggest that Nrp1 is critical for MaSCs *in vitro* colony formation.

**Figure 3:**
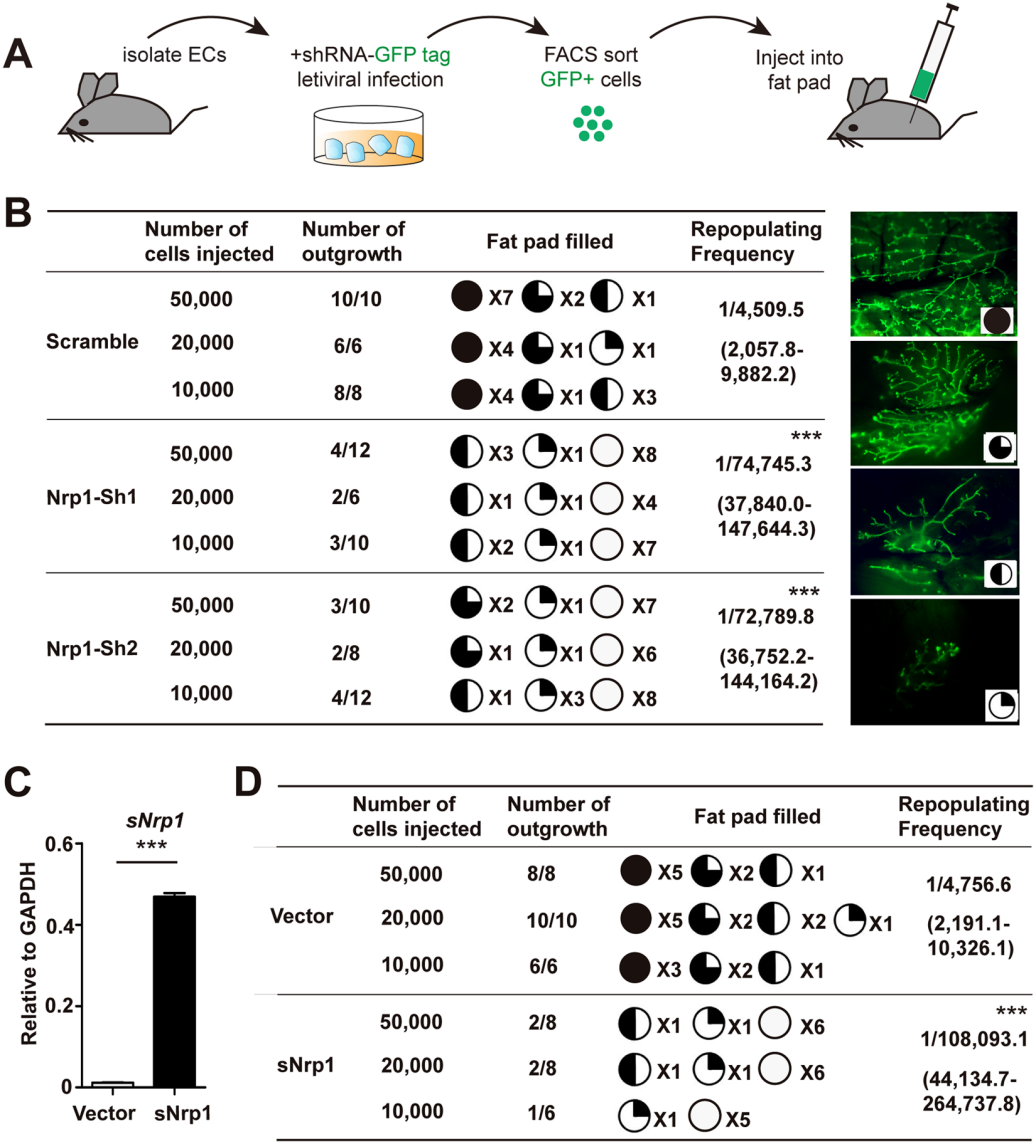
Inhibition of Nrp1 attenuates MaSCs’ reconstitution ability *in vivo*. (**A**) Schematic illustration of transplantation assay setup. Mammary epithelial cells (ECs) were isolated from 8-week-old nulliparous mice, followed by infection with scramble or *Nrp1* shRNAs lentivirus. After 6 days of culture, positively infected cells (GFP^+^) were FACS isolated and transplanted into the cleared fat pad of 3-week-old nude mice and allowed for the formation of mammary outgrowth. (**B**) 10,000 or 20,000 or 50,000 of infected cells were injected into each of the cleared fat pad, and the mammary outgrowth were examined at 8 weeks post transplantation. Number of outgrowths and percentage of fat pad filled are shown. Representative images of different percentage of fat pad filled are shown on the right panels. Data are pooled from three independent experiments. (**C**) qPCR analysis of soluble *Nrp1* (*sNrp1*) expression in infected (GFP^+^) epithelial cells. (**D**) 10,000 or 20,000 or 50,000 of infected cells with vector or sNrp1 overexpression were injected into each of the cleared fat pad, and the mammary outgrowths were examined at 8 weeks post transplantation. Data were pooled from three independent experiments. Data are presented as mean ± SEM. ***P < 0.0001.

We next investigated the impact of *Nrp1* knockdown on MaSCs in transplantation assays. Mammary cells were isolated and virally infected by Nrp1 shRNA with GFP tag, the successfully infected cells were FACS-isolated by GFP expression and transplanted to cleared fat pads of recipients (illustrated in Fig. 3A). We found that knockdown of *Nrp1* by either shRNA leads to significantly decreased repopulating efficiency, reduction of fat pad filling (Fig. 3B). To determine whether the ligand binding function of Nrp1 is important for MaSCs, we utilized the extracellular domain of Nrp1, a soluble form of Nrp1 (sNrp1), which inhibits the association of Nrp1 with its ligands^21^. sNrp1 was overexpressed in MaSCs using GFP tagged lentivirus, infected cells were FACS-isolated and transplanted to cleared fat pads. Enhanced expression of *sNrp1* was confirmed by qPCR analysis (Fig. 3C). Consistently, sNrp1 overexpression resulted in reduced repopulation efficiency and fat pad filling (Fig. 3D), suggesting a decrease in MaSC numbers. Together, these data suggest that Nrp1 is critical for the regenerative capacity of MaSCs *in vivo*.

### Nrp1 is important for mammary development

**Figure 4:**
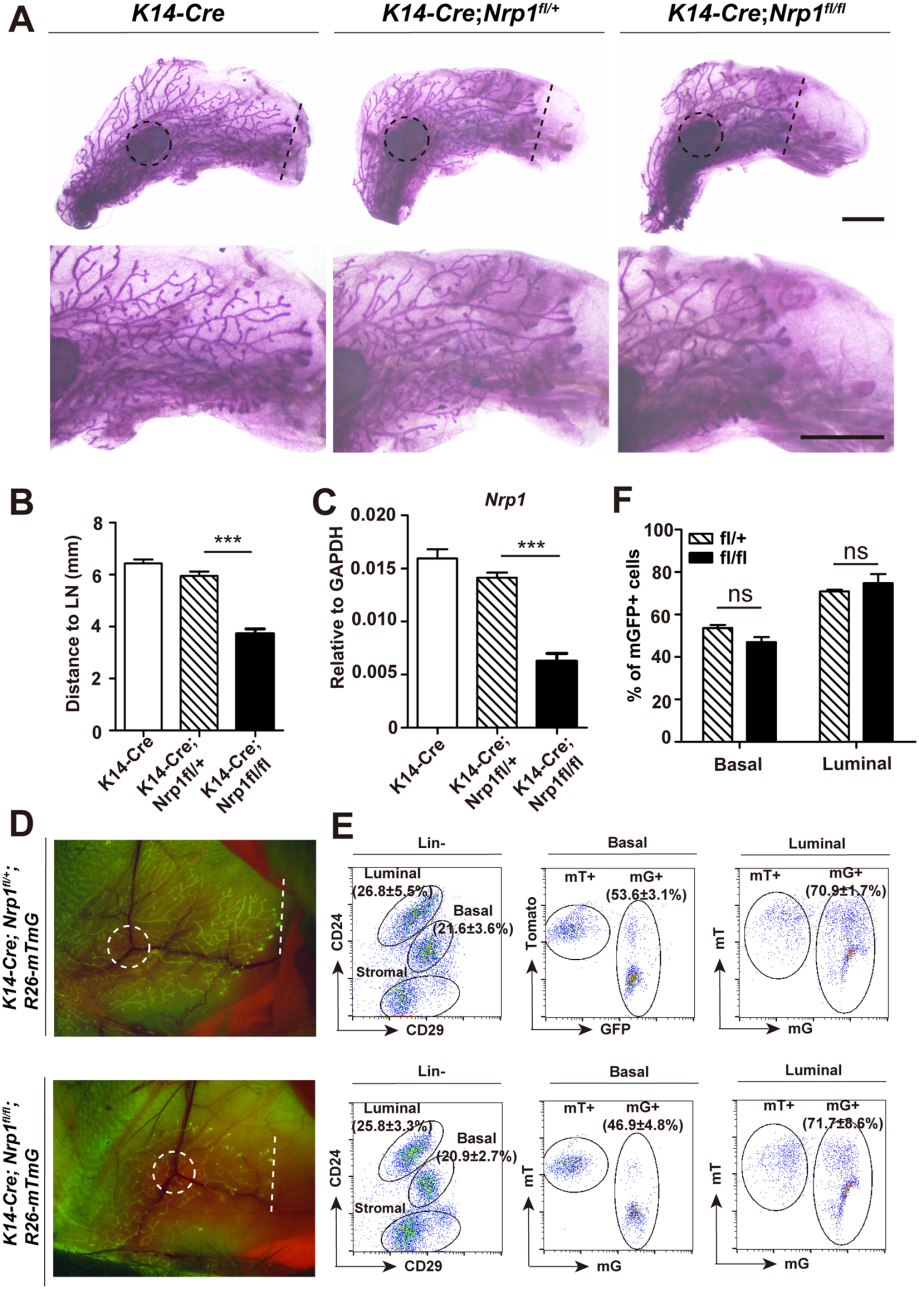
Loss of Nrp1 in basal cells compromises mammary development. (**A**) Representative images of whole mount carmine staining for 7-week old *K14-Cre*, *K14-Cre;Nrp1* ^*f*l/+^ and *K14-Cre;Nrp1* ^*f*l/*f*l^ mice. Dash line, forefront of epithelium extension; dash line circle, lymph node (L.N.). Scale bar, top panels, 2mm; bottom panels, 2mm. (**B**) Quantification of the mammary duct extension in *K14-Cre*, *K14-Cre;Nrp1* ^*f*l/+^ and *K14-Cre;Nrp1* ^*f*l/*f*l^ mice. The distance from L.N. to epithelium forefront was measured. n = 3 mice in each group. Data are presented as mean ± SEM. ***P < 0.0001. (**C**) qPCR analysis validating the *Nrp1* knockout efficiency using FACS-isolated basal cells. (**D**) Whole mount imaging of mammary glands with lineage-traced mGFP at 6-week old. (**E**,**F**) FACS analysis indicating the proportion of basal and luminal cells, and the percentages of mG+ cells in basal and luminal compartments (**E**). Quantification indicating no significant differences of mG+ cell percentage in either basal or luminal compartment when comparing the Nrp1 knockout (fl/fl) and the Ctrl (fl/+) (**F**). n = 3 mice in each group.

To investigate the function of Nrp1 in development, we utilized the Nrp1 flox allele^12^, and generated *K14-Cre*;*Nrp1* ^*f*l/*f*l^ through genetic crosses. K14 is expressed in all mammary epithelial cells during embryonic development, subsequently it is restricted in basal cells after birth. Thus, K14-Cre primarily facilitated deletion of *Nrp1* in mammary basal cells. The 4^th^ pair of mammary glands were harvested at 7-week old. We observed a delay in ductal extension in *K14-Cre*;*Nrp1* ^*f*l/*f*l^ mice, compared to *K14-Cre* and *K14-Cre;Nrp1* ^*f*l/+^ littermate controls (Fig. 4A,B). qPCR analysis in FACS-isolated basal cells confirmed the decrease of *Nrp1* expression (Fig. 4C). These results suggest that Nrp1 is important for mammary development. Next, we investigated whether Nrp1 influences lineage commitment using *K14-Cre*;*Nrp1* ^*f*l/*f*l^;*R26-mTmG* mice. The expressed Cre recombinase in basal cells would result in excision of the stop cassette in the reporter, thus marking the basal cells and their progeny, including luminal cells, with membrane-bound GFP (mG). The mammary glands were harvested at 6-week old. In whole mount imaging, consistently we observed a delayed ductal extension phenotype in *K14-Cre*;*Nrp1* ^*f*l/*f*l^;*R26-mTmG* when compared to the control (Fig. 4D). FACS analysis of the isolated primary cells indicated that 46.9% of basal cells and 71.7% of luminal cells expression mG (Fig. 4E). There were no significant differences of mG^+^ cell percentage compared to the control (Fig. 4E,F), suggesting that Nrp1 deletion does not affect basal luminal cell fate commitment.

**Figure 5:**
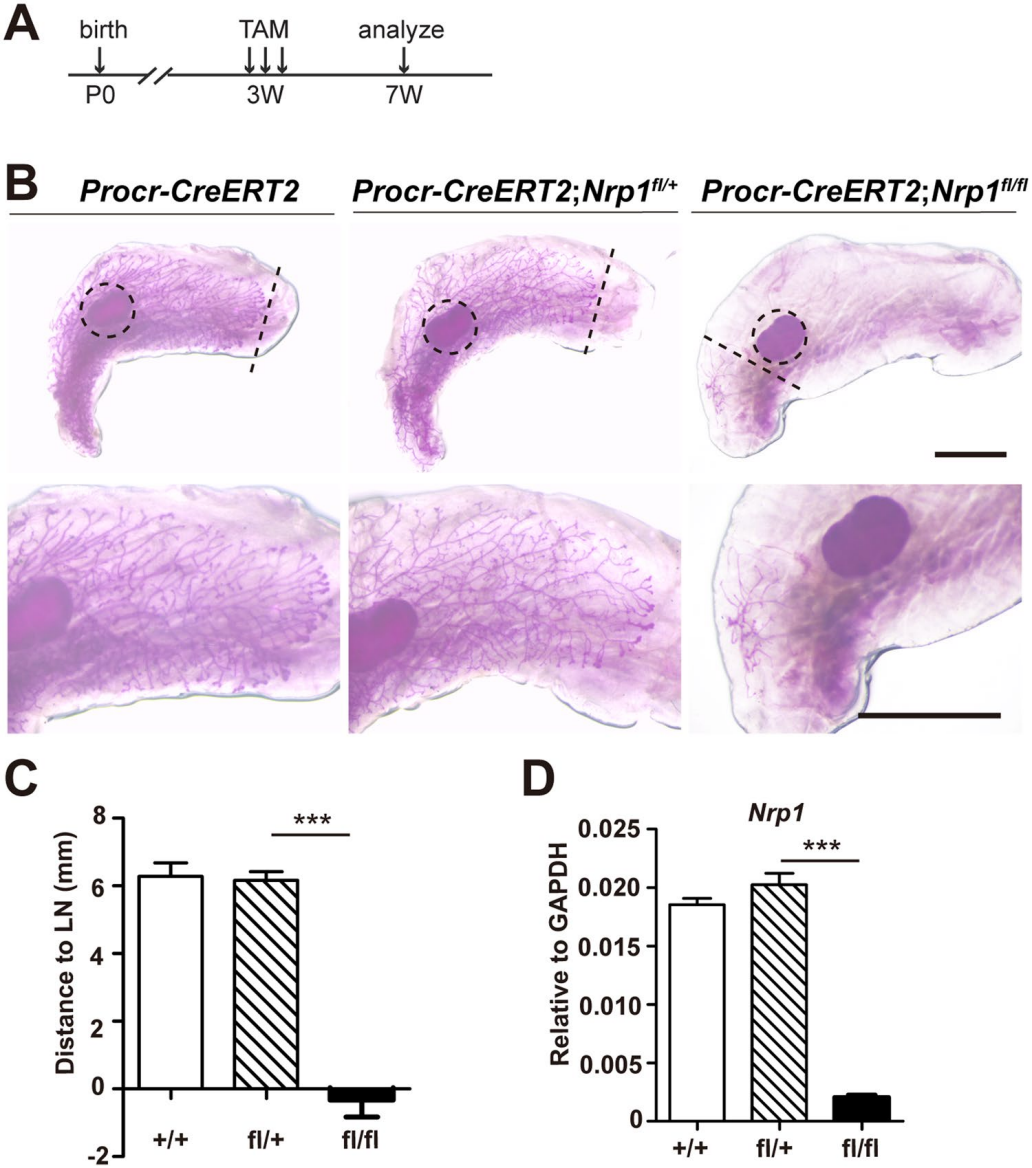
Loss of Nrp1 in MaSCs severely delays mammary development. (**A**) Experimental setup for *Nrp1* conditional knockout using *Procr-CreERT2* line. TAM was administrated at 3-week at every other day for a total of three times, mammary glands were harvest at 7-week. (**B**) Whole mount carmine staining of 7-week old *Procr-CreERT2*, *Procr-CreERT2;Nrp1* ^*f*l/+^ and *Procr-CreERT2*;*Nrp1* ^*f*l/*f*l^ mammary glands. Dash line, epithelial front; dash line circle, LN. Scale bars, 2 mm. (**C**) Quantification of the mammary duct extension in *Procr-CreERT2* (+/+), *Procr-CreERT2*;*Nrp1* ^*f*l/+^ (fl/+) and *Procr-CreERT2*;*Nrp1* ^*f*l/*f*l^ (fl/fl) mice. The distance from L.N. to epithelium forefront was measured. n = 4 mice in each group. Data are presented as mean ± SEM. ***P < 0.0001. (**D**) qPCR analysis validating the Nrp1 knockout efficiency in FACS-isolated basal cells. Data are presented as mean ± SEM. ***P < 0.0001.

Towards a more specific deletion of *Nrp1* in MaSCs, we generated *Procr-CreERT2*;*Nrp1* ^*f*l/*f*l^ mice. Tamoxifen (TAM) was administered in pubertal mice at 3-week old every other day for 3 times, the phenotype resulted from *Nrp1* deletion was evaluated at 4 weeks later (Fig. 5A). At 7-week old, the TAM-treated control mammary glands (*Procr-CreERT2* and *Procr-CreERT2*;*Nrp1* ^*f*l/+^) had almost completed the epithelium extension and occupied the whole fat pad (Fig. 5B). Strikingly, deletion of Nrp1 in *Procr-CreERT2*;*Nrp1* ^*f*l/*f*l^ mice completely prevented the growth of the epithelium: the forefront of the epithelium halted at a position where the forefront was at the initiation of *Nrp1* deletion (at around the lymph node) (Fig. 5B,C). qPCR analysis in FACS-isolated basal cells confirmed the decrease of *Nrp1* expression (Fig. 5D). These data reinforced that Nrp1 plays an important functional role in MaSC and its deficiency affects mammary development.

### Nrp1 knockdown inhibits MMTV-Wnt1 tumor growth

**Figure 6:**
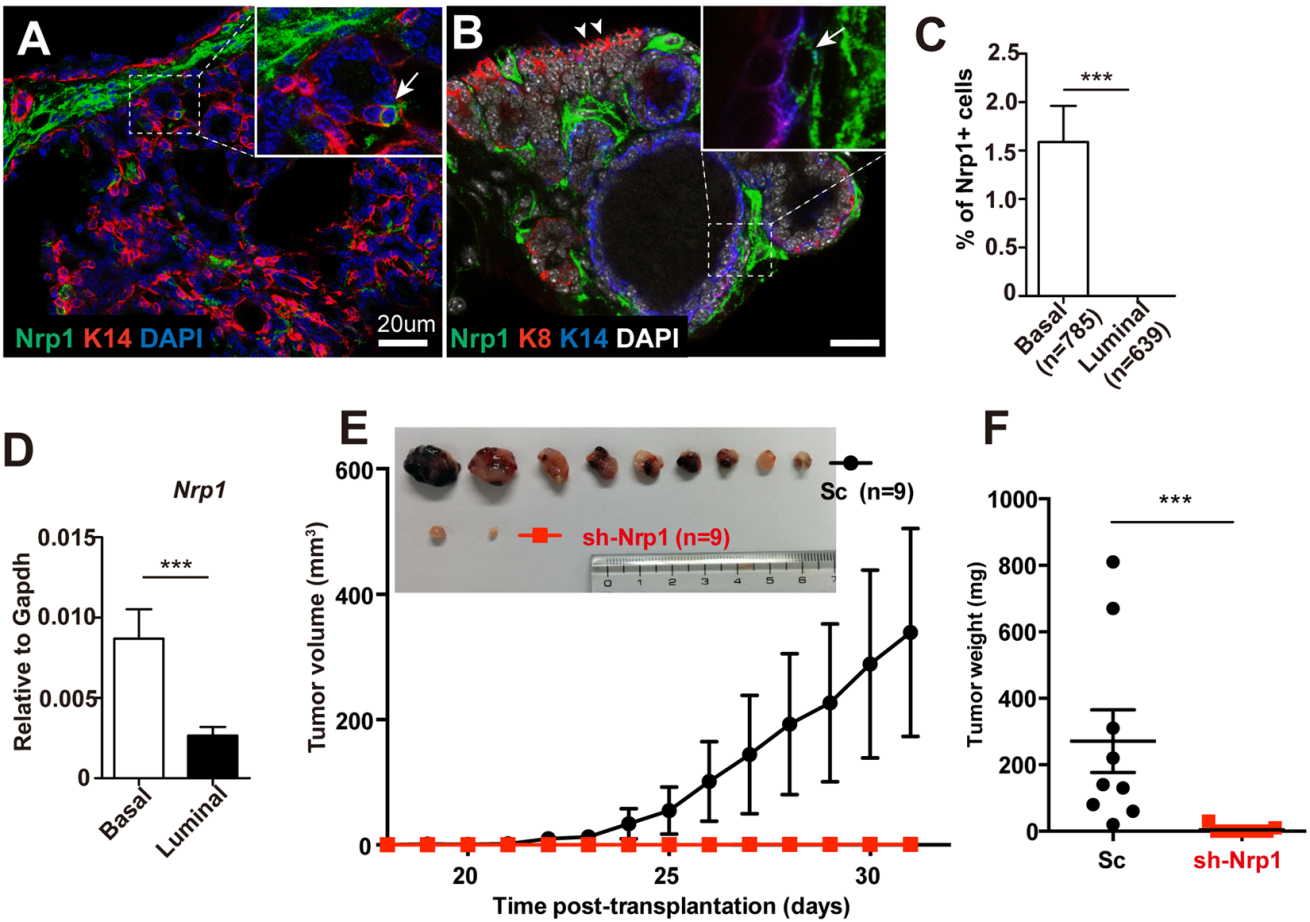
Knockdown of Nrp1 inhibits *MMTV-Wnt1* tumor growth. (**A**–**C**) Immunostaining indicating the expression of Nrp1 (green) in *MMTV-Wnt1* tumor sections. Basal cells were marked by K14 expression (red in A, blue in B), luminal cells were marked by K8 (red in B). Nrp1^+^K14^+^ cells were indicated by arrows (**A** and **B**). K8+ cells were indicated by arrowheads (**B**). The percentages of Nrp1^+^ cells were quantified in basal and luminal populations (**C**). Scale bar, 20 μm. Data are presented as mean ± SEM. ***P < 0.0001. (**D**) qPCR analysis of FACS-isolated basal and luminal cells in the tumor indicating Nrp1 expression is higher in basal cells. Data are pool from three independent experiments and presented as mean ± SEM. ***P < 0.0001. (**E**,**F**) Primary cells were isolated from *MMTV-Wnt1* tumor and infected with scramble (Sc) or *Nrp1* shRNA (*sh-Nrp1*) lentivirus. After 6 days of culture, infected cells were FACS-sorted and injected into fat pad of 8-week-old nude mice. Tumor volume was measured as indicated (**E**). Inserted image showing the tumors harvested at day 31 post surgery (**E**). Tumor weights were measured at day 31 post surgery (**F**). Data are pooled from three independent experiments and presented as mean ± SEM. ***P < 0.0001.

*MMTV-Wnt1* mammary tumor is a malignancy that has close association with MaSCs^2, 22^. Considering the important role of Nrp1 in MaSCs, we examined its impact in *MMTV-Wnt1* tumor growth. Immunofluoescent staining in tumor section suggested a sporadic pattern of Nrp1^+^ cells in *MMTV-Wnt1* tumor epithelial compartment (Fig. 6A). Immunostaining of lineage marker suggested that Nrp1^+^ cells are exclusively expressed in K14^+^ basal cells (Fig. 6A–C). qPCR analysis of FACS-isolated tumor cells confirmed the Nrp1 expression pattern (Fig. 6D). To investigate the role of Nrp1 in tumorigenesis, isolated *MMTV-Wnt1* mammary tumor cells were infected by Nrp1 shRNA. The infected cells that are marked by GFP expression were engrafted to the fat pad of nude recipients. We found that Nrp1 knockdown drastically inhibits tumor formation and attenuates tumor growth, when compared with the scramble control (Fig. 6E,F). These data suggest that Nrp1 plays an important role in *MMTV-Wnt1* tumor growth.

## Discussion

Nrp1 have been studied extensively for their roles in neural development and to some extent vascular development, but their potential contribution to epithelial tissues has not been investigated extensively. In this study, we investigated the functional role of Nrp1 in mammary development. We revealed the connection of Wnt/β-catenin signaling and Nrp1 expression in the mammary gland. In the epithelium compartment, Nrp1 was mainly expressed in Procr^+^ MaSCs. *In vitro* colony formation and *in vivo* transplantation experiments demonstrated that Nrp1 is critical for the activity of MaSCs. We provided genetic evidence that deletion of Nrp1 compromises mammary development. Furthermore, inhibition of Nrp1 attenuated *MMTV-Wnt1* mammary tumor growth.

The specific Nrp1 expression in Procr^+^ MaSCs was suggested by FACS analysis with elaborated surface markers and qPCR analysis, and further confirmed by immunofluorescent staining. Nrp1 was highly expressed in mesenchymal cells surrounding the mammary epithelium, while Nrp1 in the epithelial compartment was mainly expressed in Procr^+^ basal cells. Considering Procr^+^ MaSCs are localized in ducts, and are excluded from the TEBs in development mammary gland^4^, it is logical that Nrp1 expression would not be found in TEB epithelium. These analysis in high resolution and conclusions are contrary to a previous report, in which individual TEB or duct segments were isolated under dissecting microscope, followed by microarray and qPCR analysis, which led to a conclusion that Nrp1 is equally expressed in both TEB and duct^23^. Consider the abundant Nrp1 expression in surrounding mesenchymal cells revealed in current study, we believe that the Nrp1 expression detected from isolated mammary fragments (comprising both epithelium and mesenchyme) are most likely contributed by the Nrp1 in mesenchymal compartment.

Nrp1 has a structural homolog, Nrp2, with 44% amino acid identity. They differ in their regulation and function (reviewed in ref. 11). Nrp2 has also been reported to contribute towards branching morphogenesis of mammary epithelial cells^24^. Nrp2 is expressed preferentially in the TEBs of developing glands. Deletion of Nrp2 using *MMTV-Cre* results in defects in branching morphogenesis^24^. VEGF165, an isoform of VEGF, induces Nrp2-dependent branching morphogenesis as demonstrated *in vitro* 3D culture model with a mouse mammary cell line^24^. In this study, we found that Nrp1 is particularly expressed in Procr^+^ MaSCs, distinct from the TEB enriched expression of Nrp2; Nrp1 is a Wnt-target gene in the mammary epithelium while Nrp2 is not (data not shown). Our data demonstrated the critical role of Nrp1 for the activities of MaSCs and the development of mammary gland, yet the mechanism by which Nrp1 functions in MaSC remains to be elucidated. Mammary epithelial cells express VEGF^24^, implying a possibility that VEGF is a potential ligand for Nrp1 in MaSCs. Considering that Nrps function as co-receptor for other growth factor, including fibroblast growth factor (FGF) family^25^, galectin-1^26^, hepatocyte growth factor/scatter factor (HGF/SF)^27–29^, transforming growth factor-β1 (TGF-β1)^30, 31^, platelet-derived growth factor (PDGF)^32^, and integrin receptors^33^, thus it is possible that Nrp1 also mediates the impact of other ligands in MaSCs.

Excessive expression of both NRPs has been detected in breast cancer cells^19, 34^, associated with poor prognosis^35, 36^. Overexpression of NRPs is shown to enhance growth and correlate with invasion in various tumor types, including breast cancer^18–20, 36, 37^. In this study, our data demonstrated that Nrp1 is expressed in MaSCs of normal mammary tissues. Knockdown of Nrp1 expression in *MMTV-Wnt1* inhibited the growth of mammary tumors. Our findings reinforced the close association between *MMTV-Wnt1* mammary tumor and MaSCs^2, 22^, and suggested Nrp1 as a target to inhibit this type of breast cancer.

In conclusion, our results demonstrated the upregulation of Nrp1 by Wnt/β-catenin signaling in the mammary epithelium, exhibited the specific expression of Nrp1 in MaSCs, and revealed its critical functional roles in mammary development and tumorigenesis. Results presented in this study reinforce the connection of Wnt/β-catenin signaling and the function of MaSC, providing new insight into targeting stem cells in breast cancer through antagonizing Nrp1.

## Experimental Material and Procedures

### Experimental mice

The *K14-Cre*, *Procr-CreERT2-IRES-tdTomato*, *Nrp1* ^*flox*^ and *MMTV-Wnt1* mice were used. *Nrp1* ^*flox*^ mice were purchased from Jax (005247). All experimental mice were treated according to the protocols approved by the Animal Care and Use Committee of Shanghai Institute of Biochemistry and Cell Biology, Chinese Academy of Sciences.

### Isolation of primary mammary cells

The fat pads were isolated from 8-week-old nulliparous or other specified-stage female mice. Fat pads were minced and placed in appropriate volume (1 g/10 ml) digestion mix (RPMI 1640 with 25 mM HEPES, 5% fetal bovine serum, 1% penicillin-streptomycin-glutamine, 300 U/ml type 3 collagenase (Worthington) and digested for 2 h in a 100 rpm/min shaker at 37 °C, give a good shake every 15 min. Cells were pelleted and treated with red blood lysis buffer (Sigma) for 5 min, single cell suspension was obtained by subsequential incubation with 0.25% trypsin-EDTA at 37 °C for 5 min and 0.1 mg/ml DNase I (Sigma) for 5 min with gentle pipetting, and then filtrated through 70-μm cell strainer.

### Cell labeling and flow Cytometry

For cell labeling, the following antibodies were used: FITC conjugated CD31, CD45, TER119 (BD Pharmingen); CD24-PE-Cy7, CD29-APC (Biolegend), Procr-PE (eBioscience). Antibody incubation was performed on ice for 15 min in PBS with 5% fetal bovine serum. Before the FACS sorting, cells were filtrated through 45-μm cell strainer, and cells were sorted by using a FACSJazz (BD).

### Preparation of vectors and lentivirus

To construct shRNA vectors, the annealed oligonucleotides were ligated to pLKO.1-EGFP, which was modified by replacement of the puromycin-resistance gene with EGFP. The target sequences of shRNAs were listed: Scramble shRNA: 5′-TCCTAAGGTTAAGTCGCCCTCG-3′; Nrp1 sh1: 5′-TGGCTGCAAGATAACAGATTA-3′; Nrp1 sh2: 5′-CCAGAGAATCATGATCAACTT-3′. The extracellular domain of mouse Nrp1 (NM_008737.2) were amplified by PCR from E14.5 cDNA, and then cloned into pHIV-EGFP to overexpress sNrp1 proteins. Recombinant lentiviral particles were produced in HEK293 cells by a polyethylenimine, linear (MW 25,000, Polysciences) transfection system. Lentiviral particles were concentrated 38-fold by ultracentrifugation (Beckman Coulter) for 2 hours at 27,000 rpm, 4 °C.

### Colony formation assay

FACS sorted cells were infected with lentivirus in a ultralow attachment cluster at a density of 3 × 10^5^ cells/ml overnight, and resuspended at a density of 4 × 10^5^ cells/ml in chilled 100% growth-factor-reduced Matrigel (BD Bioscience), 50 μl/well of 24 wells cluster. The mixture was plated in 37 °C incubator for polymerization before being cultured with growth medium (DMEM/F12 1:1, 50 ng/ml EGF (Sigma) and ITS (1:100; Sigma)) which was changed the other day. Primary colony numbers were counted and the colony sizes were measured after 6 days culture. For passaging colonies, the medium was aspirated, and Matrigel was incubated in 500 μl of dispase (BD Bioscience) for 1 h  at 37 °C. Colonies were released from Matrigel and single cells were obtained by incubation in 0.05% Trypsin-EDTA for 5–10 min at 37 °C. Single cells were then resuspended in Matrigel as described above at the same density.

### EdU labelling

Half of *in vitro* culture colony growth medium was aspirated, replaced with growth medium containng 0.2 mg/ml EdU (Life Technologies), followed experiment steps were performed according to the manufacturer’s protocol of Click-it EdU imaging kit with Alexa Fluo 594 (Life Technologies) prior to immunohistochemistry detection of EGFP.

### Quantitative RT-PCR

Total RNA was extracted with RNAiso plus (Takara) or mini RNA preparation kit (Qiagen), and the reverse transcription reaction was performed by using PrimeScript RT Master mix kit (Takara). Quantitative RT-PCR was performed on StepOne Plus (Applied Biosystems) with FastStart Universal SYBR Green Master Mix kit (Roche). The RNA levels were analyzed by ΔΔCt method and normalized to Gapdh. qPCR product specificity was determined by melt curve analysis. The primers using in this study were listed:

Gapdh-F: TGTGATGGGTGTGAACCACGAGAA

Gapdh-R: CTGTGGTCATGAGCCCTTCCACAA

Axin2-F: AGCCTAAAGGTCTTATGTGGCTA

Axin2-R: ACCTACGTGATAAGGATTGACT

Nrp1-F: ACAAATGTGGCGGGACCATA

Nrp1-R: CCGGAGCTTGGATTAGCCAT

Procr-F: CTCTCTGGGAAAACTCCTGACA

Procr-R: CAGGGAGCAGCTAACAGTGA

### ChIP-qPCR

Comma D β cells (2 × 10^6^) were plated for 48 h and treated with vehicle or Wnt3A protein for 24 h before PFA crosslinking. Briefly, cells were cross-linked for 10 min with final concentration 1% PFA at room temperature. 125 mM final glycin was added to inactivate PFA at 37 °C for 5 min. Cells were scrapped and pelleted, cell pellet was incubated in chilled Mg-NI NP40 buffer for 10 min, then resuspended in lysis buffer with proteinase inhibitors. Genomic DNA was sonicated. Precleared sample (0.1 ml) was saved to assess input DNA. Anti-β-catenin antibody (4 μg, BD Pharmingen) was added to the precleared sample and gently mixed for O/N at 4 °C, followed by rotation with 50 μl protein G-agarose/Salmon sperm DNA (Millipore) for 2 h at 4 °C. Subsequent wash, elution, proteinase K treatment and de-cross-linking procedures were performed. TBEs enrichment was determined by qPCR analysis.

### Immunohistochemistry

Frozen sections were prepared by air-drying and fixation for 1 h in pre-chilled 4% PFA. Tissue sections were blocked with PBT contained 10% normal goat or cow serum for 1 h, then incubated with primary antibodies at 4 °C overnight, followed by PBT wash 3 times, incubated with secondary antibodies for 2 h at room temperature, and counterstained with DAPI (Life Technologies) for 2 min, mounted with prolong gold mounting medium. The primary antibodies used in this work were goat anti-mouse Nrp1 (R&D systems, AF566, 1:100), Rat anti-K8 (Developmental Hybridoma Bank, TROMA-I, 1:200) and rabbit anti-mouse Keratin 14 (Covance, PRB-155P, 1:1000). IHC detection was performed with appropriate secondary conjugates (Life Technologies). Confocal images were captured by using Leica DM6000 TCS/ SP8 laser confocal scanning microscope.

### Mammary gland whole mount carmine staining

The 4^th^ pair of mammary glands were fixed in pre-chilled 4% PFA and washed with PBS twice. Tissues were incubated with carmine alum overnight, followed by destaining with solutions (50% ethanol, 2% HCl) for 2 h, dehydrated in 70%, 85%, 95%, 100% ethanol for 1 h, and treated with histoclear for 1 h. Images were captured by a dissection microscope (Leica).

### Transplantation into mammary fat pad

FACS Sorted cells were resuspended in 50% matrigel, PBS with 20% FBS, and 0.04% Trypan Blue (Sigma) and injected 10 μl volumes into the cleared fat pads of 3-week-old female nude mice. Reconstituted mammary glands were harvested 8 weeks after transplantation. Outgrowths were detected under a dissection microscope (Leica) based on fluorescence or carmine staining. Outgrowths with >10% of the host fat pad filled were scored as positive, and fat pad filled was marked as 4/4 (all) filled, 3/4, 1/2, 1/4 and no growth. The repopulating frequencies were calculated using the “statmod” software package for the R computing environment (http://www.R-project.org). Repopulating frequencies were estimated with a complementary log-log generalized linear model. Two-sided 95% Wald confidence intervals were computed, except in the case of zero outgrowths, when one-sided 95% Clopper-Pearson intervals were used instead.

### Statistical analysis

Student’s t-test was performed and the *P* value was calculated in Graphpad Prism 5 on data represented by histograms, which consisted of results from at least three independent experiments unless specified otherwise. For all experiments with error bars, the standard deviation (SD) was calculated to indicate the variation within each experiment. *P* < 0.05 (*), *P* < 0.01 (**) and *P* < 0.001 (***) were considered as significance.

## References

[1] Yu, Q. C., Verheyen, E. M. & Zeng, Y. A. Mammary Development and Breast Cancer: A Wnt Perspective. *Cancers (Basel)* **8**, doi:10.3390/cancers8070065 (2016). 10.3390/cancers8070065 PMC4963807 27420097

[2] Shackleton M (2006). Generation of a functional mammary gland from a single stem cell. Nature.

[3] Stingl J (2006). Purification and unique properties of mammary epithelial stem cells. Nature.

[4] Wang D (2015). Identification of multipotent mammary stem cells by protein C receptor expression. Nature.

[5] Wend P, Holland JD, Ziebold U, Birchmeier W (2010). Wnt signaling in stem and cancer stem cells. Semin Cell Dev Biol.

[6] Incassati A, Chandramouli A, Eelkema R, Cowin P (2010). Key signaling nodes in mammary gland development and cancer: beta-catenin. Breast cancer research: BCR.

[7] Roarty K, Rosen JM (2010). Wnt and mammary stem cells: hormones cannot fly wingless. Current opinion in pharmacology.

[8] Zeng YA, Nusse R (2010). Wnt proteins are self-renewal factors for mammary stem cells and promote their long-term expansion in culture. Cell Stem Cell.

[9] Cai C (2014). R-spondin1 is a novel hormone mediator for mammary stem cell self-renewal. Genes Dev.

[10] Fujisawa H (1997). Roles of a neuronal cell-surface molecule, neuropilin, in nerve fiber fasciculation and guidance. Cell and tissue research.

[11] Wild JR, Staton CA, Chapple K, Corfe BM (2012). Neuropilins: expression and roles in the epithelium. Int J Exp Pathol.

[12] Gu C (2003). Neuropilin-1 conveys semaphorin and VEGF signaling during neural and cardiovascular development. Dev Cell.

[13] Geretti E, Shimizu A, Klagsbrun M (2008). Neuropilin structure governs VEGF and semaphorin binding and regulates angiogenesis. Angiogenesis.

[14] Kolodkin AL (1997). Neuropilin is a semaphorin III receptor. Cell.

[15] Kitsukawa T, Shimono A, Kawakami A, Kondoh H, Fujisawa H (1995). Overexpression of a membrane protein, neuropilin, in chimeric mice causes anomalies in the cardiovascular system, nervous system and limbs. Development.

[16] Kawasaki T (1999). A requirement for neuropilin-1 in embryonic vessel formation. Development.

[17] Giger RJ (2000). Neuropilin-2 is required *in vivo* for selective axon guidance responses to secreted semaphorins. Neuron.

[18] Bachelder RE (2001). Vascular Endothelial Growth Factor Is an Autocrine Survival Factor for Neuropilin-expressing Breast Carcinoma Cells. Cancer Res.

[19] Bachelder RE (2003). Competing autocrine pathways involving alternative neuropilin-1 ligands regulate chemotaxis of carcinoma cells. Cancer Res.

[20] Barr MP (2005). A peptide corresponding to the neuropilin-1-binding site on VEGF(165) induces apoptosis of neuropilin-1-expressing breast tumour cells. Br J Cancer.

[21] Gagnon ML (2000). Identification of a natural soluble neuropilin-1 that binds vascular endothelial growth factor: *In vivo* expression and antitumor activity. Proc Natl Acad Sci USA.

[22] Li Y (2003). Evidence that transgenes encoding components of the Wnt signaling pathway preferentially induce mammary cancers from progenitor cells. Proc Natl Acad Sci USA.

[23] Morris JS (2006). Involvement of axonal guidance proteins and their signaling partners in the developing mouse mammary gland. J Cell Physiol.

[24] Goel HL (2011). Neuropilin-2 promotes branching morphogenesis in the mouse mammary gland. Development.

[25] West DC (2005). Interactions of multiple heparin binding growth factors with neuropilin-1 and potentiation of the activity of fibroblast growth factor-2. J Biol Chem.

[26] Hsieh SH (2008). Galectin-1, a novel ligand of neuropilin-1, activates VEGFR-2 signaling and modulates the migration of vascular endothelial cells. Oncogene.

[27] Matsushita A, Gotze T, Korc M (2007). Hepatocyte growth factor-mediated cell invasion in pancreatic cancer cells is dependent on neuropilin-1. Cancer Res.

[28] Hu B (2007). Neuropilin-1 promotes human glioma progression through potentiating the activity of the HGF/SF autocrine pathway. Oncogene.

[29] Sulpice E (2008). Neuropilin-1 and neuropilin-2 act as coreceptors, potentiating proangiogenic activity. Blood.

[30] Cao S (2010). Neuropilin-1 promotes cirrhosis of the rodent and human liver by enhancing PDGF/TGF-beta signaling in hepatic stellate cells. The Journal of clinical investigation.

[31] Glinka Y, Prud’homme GJ (2008). Neuropilin-1 is a receptor for transforming growth factor beta-1, activates its latent form, and promotes regulatory T cell activity. Journal of leukocyte biology.

[32] Ball SG, Bayley C, Shuttleworth CA, Kielty CM (2010). Neuropilin-1 regulates platelet-derived growth factor receptor signalling in mesenchymal stem cells. The Biochemical journal.

[33] Shintani Y, Takashima S, Kato H, Komamura K, Kitakaze M (2009). Extracellular protein kinase CK2 is a novel associating protein of neuropilin-1. Biochemical and biophysical research communications.

[34] Stephenson JM, Banerjee S, Saxena NK, Cherian R, Banerjee SK (2002). Neuropilin-1 is differentially expressed in myoepithelial cells and vascular smooth muscle cells in preneoplastic and neoplastic human breast: A possible marker for the progression of breast cancer. Int J Cancer.

[35] Yasuoka H (2009). Neuropilin-2 expression in breast cancer: correlation with lymph node metastasis, poor prognosis, and regulation of CXCR4 expression. BMC cancer.

[36] Ghosh S (2008). High levels of vascular endothelial growth factor and its receptors (VEGFR-1, VEGFR-2, neuropilin-1) are associated with worse outcome in breast cancer. Human pathology.

[37] Timoshenko AV, Rastogi S, Lala PK (2007). Migration-promoting role of VEGF-C and VEGF-C binding receptors in human breast cancer cells. Br J Cancer.

